# Liver Transplantation *Versus* Liver Resection for Stage I and II Hepatocellular Carcinoma: Results of an Instrumental Variable Analysis

**DOI:** 10.3389/fonc.2021.592835

**Published:** 2021-05-26

**Authors:** Wei Li, Haitao Xiao, Hong Wu, Xuewen Xu, Yange Zhang

**Affiliations:** ^1^ Department of Plastic and Burns Surgery, West China Hospital, Sichuan University, Chengdu, China; ^2^ Department of Liver Surgery & Liver Transplantation Center, West China Hospital, Sichuan University, Chengdu, China

**Keywords:** hepatocellular carcinoma, liver resection, liver transplantation, survival, instrumental variable analysis

## Abstract

**Background:**

This study aimed to compare the long-term outcomes of liver transplantation (LT) and liver resection (LR) among patients with stage I and II hepatocellular carcinoma (HCC).

**Methods:**

SEER 18 registry from 2004 to 2015 was retrieved for this study. We included 1,765 and 1,746 cases with stage I–II (AJCC, 7^th^) HCC in the multivariable analyses and instrumental variable (IV) analyses, respectively. Propensity score matching (PSM) was further carried out to ensure comparability. Propensity score to receive LT was adjusted by stabilized inverse probability of treatment weighting (IPTW) and standardized mortality ratio weighting (SMRW) methods. In addition, IV analysis was performed to adjust both measured and unmeasured confounding factors.

**Results:**

We identified 1,000 (56.7%) and 765 (43.3%) patients treated with LR and LT, respectively. In the multivariable adjusted cohort, after adjusting potential confounders, patients undergoing LT offered significant prognostic advantages over LR in overall survival (OS, P < 0.001) and disease-free survival (DSS, P < 0.001). The instrument variable in this study is LT rates in various Health Service Areas (HSAs). Results from the IV analysis showed that cases treated with LT had significantly longer OS (P = 0.001) and DSS (P < 0.001). In IV analysis stratified by clinicopathologic variables, the treatment effect of LT *vs.* LR in OS was consistent across all subgroups. Regarding DSS in IV analyses, the subgroup analyses observed that LT had better DSS across all subgroups, except for similar results in the older patients (interaction P value = 0.039) and the non-White patients (interaction P value = 0.041). In the propensity-matched cohort, patients with LT still had better OS (P < 0.001) and DSS (P < 0.001) in comparison to cases who underwent LR. In both IPTW and SMRW cohorts, patients who underwent LT had better OS (both P values < 0.001) and DSS (both P values < 0.001).

**Conclusions:**

LT provided a survival benefit for cases with stage I–II HCC. These results indicated that if LT rate was to increase in the future, average long-term survival may also increase. However, for some special populations such as the elderly patients, owing to the similar outcomes between LT and LR, the selection of LT should be cautious.

## Introduction

Liver cancer is the second most frequent cause of cancer death worldwide (1). Hepatocellular carcinoma (HCC) is the most common type of primary liver cancer globally (2). Liver resection (LR) is recommended as first-line treatment in HCC patients without liver cirrhosis (1). In contrast, for HCC cases with cirrhosis, indications for LR are generally based on the comprehensive evaluation of tumor burden, liver function, extent of resection, expected remnant liver volume, cases’ comorbid conditions, and performance status (3, 4). Except for LR, liver transplantation (LT) is also an excellent radical therapy choice for HCC cases, eliminating both of the underlying liver cirrhosis and tumor. LT is a first-line therapeutic option for tumors meeting the Milan criteria but unsuitable for resection (1). Despite these recommendations, for early stage HCC patients with compensated liver function, in some situations (*e.g.*, patients with available liver donation), LT can also be utilized to achieve radical cure (5–7).

For cases with early stage HCC who are candidates for both LT and LR, there is no consensus on the eligibility criteria for LR or LT in the current data (5, 6, 8–11). Recent studies comparing LT with LR have demonstrated superior survival outcomes of LT in patients with early stage HCC (6, 12). However, owing to the significant heterogeneity among the included patients in these retrospective studies, it is still controversial with regard to which modality provides better long-term results. The aim of the present study was to compare the long-term outcomes of LT and LR in cases with early stage (stages I and II) HCC. To achieve it, instrumental variable (IV) analyses were used in this study. IV analysis is a statistical method that serves as an alternative to random assignment to treatment and addresses confounders owing to both known and unknown factors (13, 14).

## Patients and Methods

### Patient Identification

**Figure 1:**
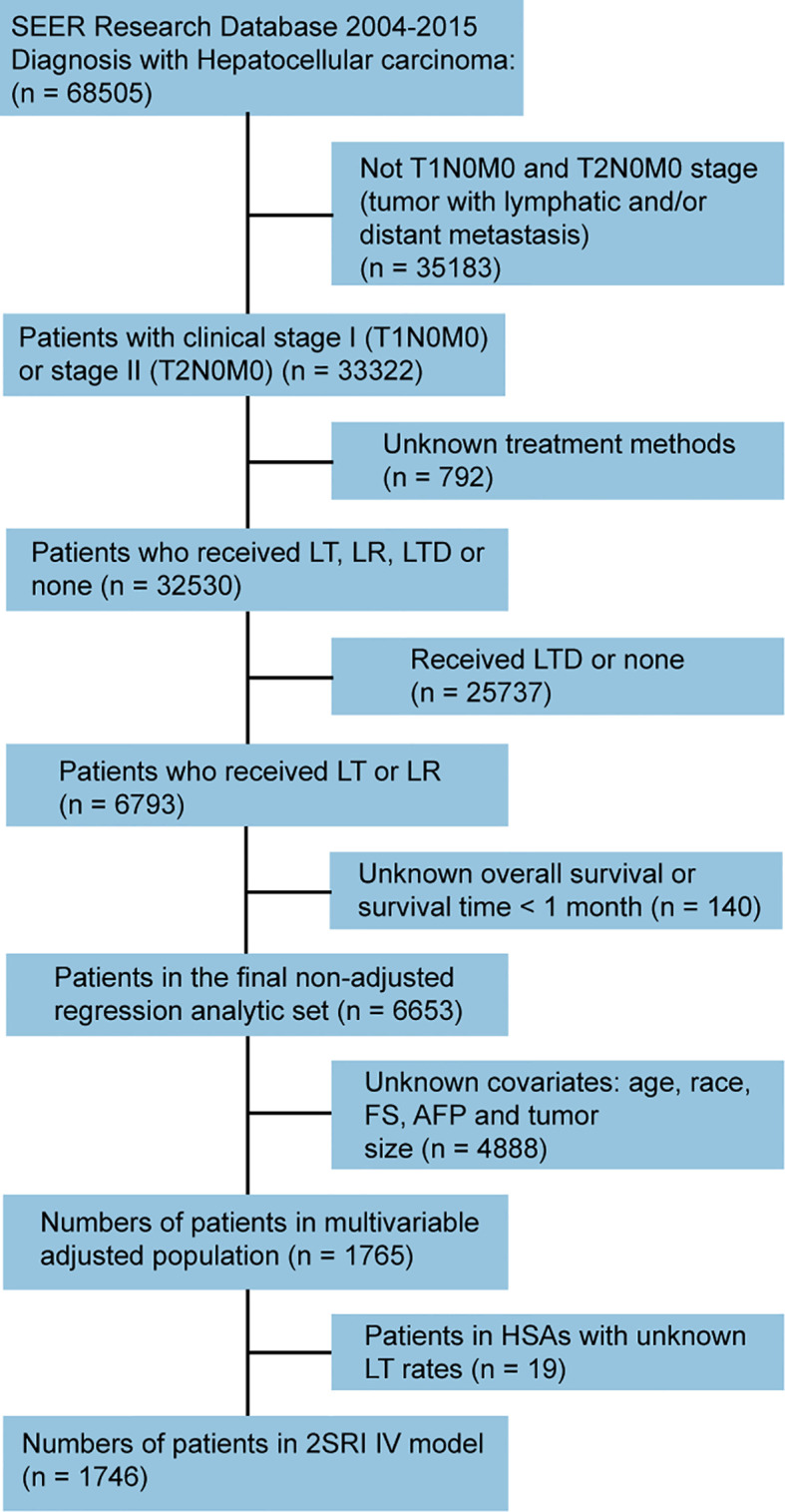
Flowchart showing selection process of cases included in this study. SEER, Surveillance, Epidemiology, and End Results; LT, liver transplantation; LR, liver resection; LTD, local tumor destruction; FS, fibrosis score; AFP, alpha-fetoprotein; HAS, Health Service Area; 2SRI, two-stage residual inclusion; IV, instrumental variable.

Surveillance, Epidemiology, and End Results (SEER; seer.cancer.gov/about/overview.html) 18 database from 2004 to 2015 was retrieved for this study. Firstly, 68505 patients with pathological diagnosis as HCC were identified according to the International Classification of Diseases for Oncology, 3rd Edition [ICD-O-3] site code C22.0 and histologic type ICD-O-3 codes 8170-8175. All cases were treated between 2004 and 2015 from the SEER database. Flowchart of the patient selection process was presented in **Figure 1**. Patients with early-stage (stage I and II; AJCC, 7th) HCC matching the specified eligibility criteria were included in the multivariable analyses (n = 1765) and IV analyses (n = 1746), respectively. The codes in SEER database for HCC treatment included: LR: 20-25, 30, 36, 37, 50, 51, and 52; LT: 61.

### Statistical Analysis

Overall survival (OS) was defined as the interval from the date of diagnosis to the date of death with any causes, and the disease-specific survival (DSS) was defined as the time until death attributed to HCC. Continuous variables were presented as mean ± SD (tested by t-test or Kruskal-Wallis H test) and categorical variables were expressed as number (%) (tested by Chi-square test or Fisher’s exact test). Linear trends in the percentage of patients receiving each type of treatment was evaluated by Cochrane-Armitage trend test.

Survival curves were performed using the Kaplan-Meier method and the differences in the survival rates between two groups were compared via log-rank test. Multivariable Cox models were used to adjust for available confounding factors. Interaction tests were used to examine the influence of each stratified indicator on the relations between surgical modality and patient prognosis.

Propensity score-matched (PSM) analysis was done based on the following factors: race, sex, age, year of diagnosis, tumor size, fibrosis-score (Ishak; FS) and alpha-fetoprotein (AFP). Cases were matched with the closest estimated propensity score within 0.02, and we performed a 1:1 nearest-neighbor matching with the preset caliber. Univariable Cox regression was utilized to compare the survival outcomes of LR vs. LT in the cohort after PSM selection.

In addition, PS to receive LT was adjusted by a standardized mortality ratio weighting (SMRW) and stabilized inverse probability of treatment weighting (IPTW) methods. The IPTW assigned weights of 1/PS for patients receiving LT and 1/ (1-PS) for patients undergoing LR. The SMRW assigned a weight of 1 for LT patients and a weight of PS/ (1-PS) for cases with LR. OS and DSS of LT vs. LR were then compared (univariable Cox regression) using the PS-adjusted pseudopopulation created by these two statistical procedures.

In this study, the LT rate in different Health Service Areas (HSAs) was utilized as the instrumental variable. The IV approach depends on the assumption that LT rate was highly related to the selection of treatment methods (cases with higher HAS LR rates usually had a higher opportunity to receive LR), and the IV was not associated with patient survival except through its correlation with the treatment methods (15). In addition, the IV was unrelated to unmeasured risk factors affecting the outcome. Cases from HSAs with less than 10 cases were excluded, because the LT rates could not be confirmed accurately in those HSAs (16). To assess the validity of LT rates in HSAs as an IV, we verified that LT rate in a HSA was significantly associated with likelihood of treatment assignment (the F statistic exceeding 10 is suggestive of a strong instrument), while not associated with OS in the Multivariable regression analysis. Besides, covariate balance was examined across quintiles. We used a two-stage residual inclusion (2SRI) method in the instrumental variable analysis (17).

It is important to note that, rather than exploring the average treatment effects for a group of cases (as in a randomized trial), the IV analysis focuses on the treatment effect among those whose selection of therapy is affected by the instrumental variable (18). LT rates in HSAs was utilized as the IV, which indicates that our results are generalizable only to cases whose treatment assignment was influenced by the LT rates in different HSAs. In summary, this study analyzed the treatment effect among marginal patients. The marginal patients are those with early-stage HCC would receive LT in a areas with higher LR rates while not in HSAs with lower LR rates, (18, 19) because treatment method (LT or LR) for cases with a uncertain or borderline need for LT could be influenced by experience and preferences in different areas. P value < 0.05 was defined as statistically significant. Statistical analysis was carried out by R 3.6.3.

## Results

### Demographics

**Figure 2:**
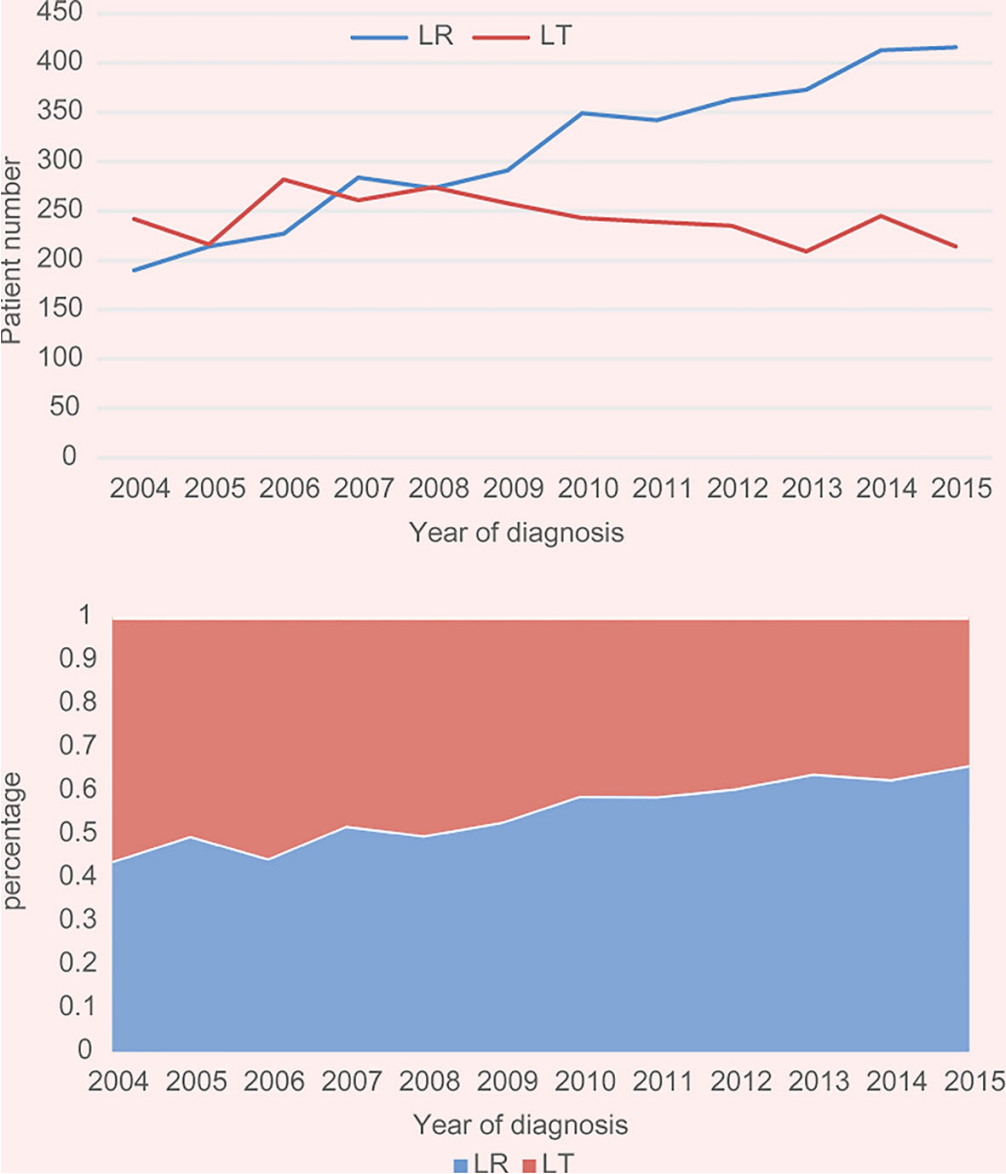
**(A)** Number of cases with stage I–II HCC from 2004 to 2015 in the SEER cohort. **(B)** Incidence of cases with stage I–II HCC from 2004 to 2015 in the SEER cohort (both P trend values for LT and LR <0.001).

Among 6653 patients treated surgically for stage I and II HCC, we identified 1000 (56.7%) and 765 (43.3%) patients treated with LR or LT, respectively. **Figure 2** showed the number and incidence of 6653 cases with stage I-II HCC (AJCC 7th) between 2004 and 2015 with LT or LR. Incidence rate of LT was decreased over time (P < 0.001), while incidence of cases undergoing LR was increased over time (P < 0.001). The general patient characteristics was shown in **Table 1**. The mean age of patients with LT and LR was 57.1 and 62.6 years, respectively. Cases undergoing LT were younger, more often male and the White, and more patients had stage II disease. When patients underwent LT, their tumors were more likely to measure < 3 cm (65.8%), and more cases had cirrhotic liver (88.9%). For cases with LR, more cases had non-cirrhotic liver (FS in 53.5% of cases was between 0-4), and 35% of cases had tumors larger than 5 cm.

**Table 1:** Characteristics of the entire study sample by treatment received.

	LR (n = 1,000)	LT (n = 765)	P value
Sex (female/male)	269/731	165/600	0.010
Age (years)			
≥18, <45	44 (4.4%)	29 (3.8%)	<0.001
≥45, <60	324 (32.4%)	427 (55.8%)	
≥60, <70	380 (38.0%)	286 (37.4%)	
>=70	252 (25.2%)	18 (2.4%)	
Marriage status (married/divorced or separated/single)	644/181/154	516/113/111	0.160
Insurance (yes/no)	867/14	593/2	0.037
Race (White/Black/other/unknown)	511/128/354/7	595/63/105/2	<0.001
Year of diagnosis (2004–2009/2010–2015)	307/693	341/424	<0.001
AFP (ng/ml negative/positive)	399/601	305/460	0.990
Tumor size (cm)			<0.001
<3	267 (26.7%)	503 (65.8%)	
≥3, <5	363 (36.3%)	225 (29.4%)	
≥5, <7	175 (17.5%)	32 (4.2%)	
≥7	195 (19.5%)	5 (0.7%)	
One lesion in one lobe (yes/no)	602/398	341/424	<0.001
Vascular invasion (no/yes)	766/234	548/217	0.018
AJCC-TNM stage (I/II/)	694/306	372/393	<0.001
Fibrosis score (0–4/5–6)	535/465	85/680	<0.001
Tumor differentiation (I/II/III/IV/unknown)	234/559/191/16/0	255/424/84/2/0	<0.001

Data are shown as mean ± SD or n (%). LR, liver resection; LT, liver transplantation; AFP, alpha-fetoprotein; AJCC, American Joint Committee on Cancer. Tumor differentiation: I, well-differentiated; II, moderate-differentiated; III, poor-differentiated; IV, un-differentiated.

### Multivariable Cox Regression

**Figure 3:**
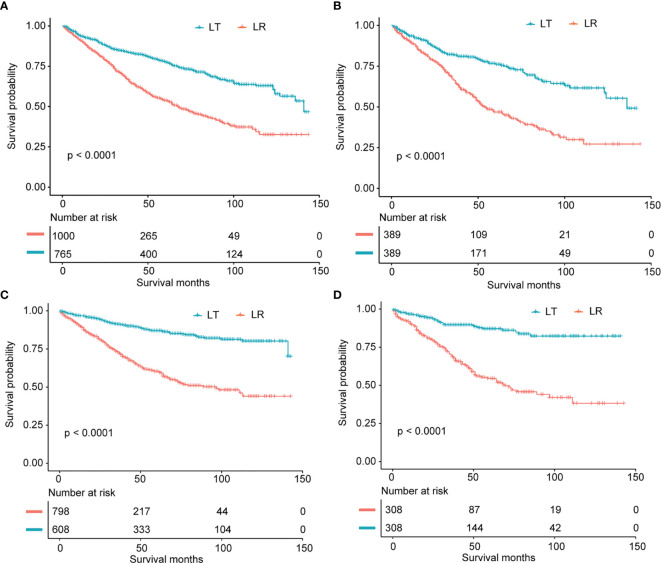
**(A)** Overall survival analysis for patients who underwent LR and LT in non-adjusted population. **(B)** Overall survival analysis for patients after LR and LT in propensity score matched cohort. **(C)** Disease-specific survival analysis for cases receiving LR and LT in non-adjusted cohort. **(D)** Disease-specific survival analysis for cases after LR and LT in propensity score matched cohort.

The current study included a total of 1765 cases with available data needed in survival analysis. The mean DSS for cases with LT or LR were 124.0 and 87.4 months, respectively. The mean OS for all of the cases receiving LT or LR were 106.6 and 77.8 months, respectively. In survival analysis, cases undergoing LT showed longer OS (P < 0.001) and DSS (P < 0.001) in comparison to cases receiving LR (**Figures 3A, C**).

The results in the multivariable adjusted cohort (OS: n = 1765; DSS: n = 1406) showed that cases receiving LT had a better DSS (HR 0.21, 95% CI 0.15 to 0.29, P < 0.001) and OS (HR 0.27, 95% CI 0.23 to 0.33, P < 0.001) compared to those undergoing LR.

### Instrumental Variable Analyses

All cases were divided into quintiles based on the proportion of patients within each HSA undergoing LT (**Supplementary Table 1**). The average LT rate ranged from 3% (quintile 1) to 8% (quintile 5) among different HSAs. The F-statistic is 104.8 (P < 0.001), which confirmed the validity of this instrument. Besides, there was no significant relationship between the IV and OS in a standard Cox regression analysis (HR 1.12, 95% CI 0.94-1.34, P = 0.198). In summary, these observations indicated that LT rate in HSAs could be utilized as a valid instrument variable. 

Finally, results in the IV analysis were consistent with those observed in the traditional regression analyses. Outcomes according to this instrument demonstrated that patients receiving LT had an obviously better DSS (HR 0.29, 95% CI 0.16-0.55, P < 0.001) and OS (HR 0.47, 95% CI 0.29-0.75, P = 0.001) after adjusting both measured and unmeasured confounders (**Table 2**).

**Table 2:** Instrumental variable analysis of the impact of surgery methods (LT *vs.* LR) on survival for patients with hepatocellular carcinoma in 2SRI IV Model.

	All-cause survival			Cancer-specific survival		
	HR	95% CI	P-value	HR	95% CI	P-value
LT *vs.* LR	0.47	0.29–0.75	0.001	0.29	0.16–0.55	<0.001
Age, years	1.02	1.01–1.03	<0.001	1.02	1.00–1.03	0.017
Sex, male *vs.* female	1.05	0.86–1.28	0.656	0.17	0.89–1.53	0.268
Race						
Black *vs.* White	1.18	0.90–1.53	0.2315	1.39	0.99–1.94	0.056
Other *vs.* White	0.70	0.57–0.87	<0.001	0.67	0.51–0.88	0.004
AFP level ng/ml, positive *vs.* negative	1.30	1.08–1.57	0.005	1.22	0.96–1.55	0.111
Tumor size, cm	1.00	1.00–1.00	<0.001	1.00	1.00–1.01	<0.001
AJCC stage, II *vs.* I	1.21	1.01–1.45	0.034	1.50	1.19–1.89	0.001
Fibrosis score, 5–6 *vs.* 0–4	1.66	1.35–2.03	<0.001	1.65	1.28–2.14	<0.001
Tumor differentiation						
Moderate-differentiated *vs.* well-differentiated	1.18	0.96–1.47	0.120	1.28	0.94–1.73	0.116
Poor-differentiated *vs.* well-differentiated	1.56	1.22–2.10	<0.001	1.92	1.34–2.76	<0.001
Un-differentiated *vs.* well-differentiated	1.58	0.77–3.27	0.216	1.83	0.78–4.33	0.168
Year of diagnosis, 2010–2015 *vs.* 2004–2009	0.97	0.96–0.99	0.001	0.97	0.95–0.99	0.003

LR, liver resection; LT, liver transplantation; AFP, alpha-fetoprotein; AJCC, American Joint Committee on Cancer.

### Stratified Analyses

**Figure 4:**
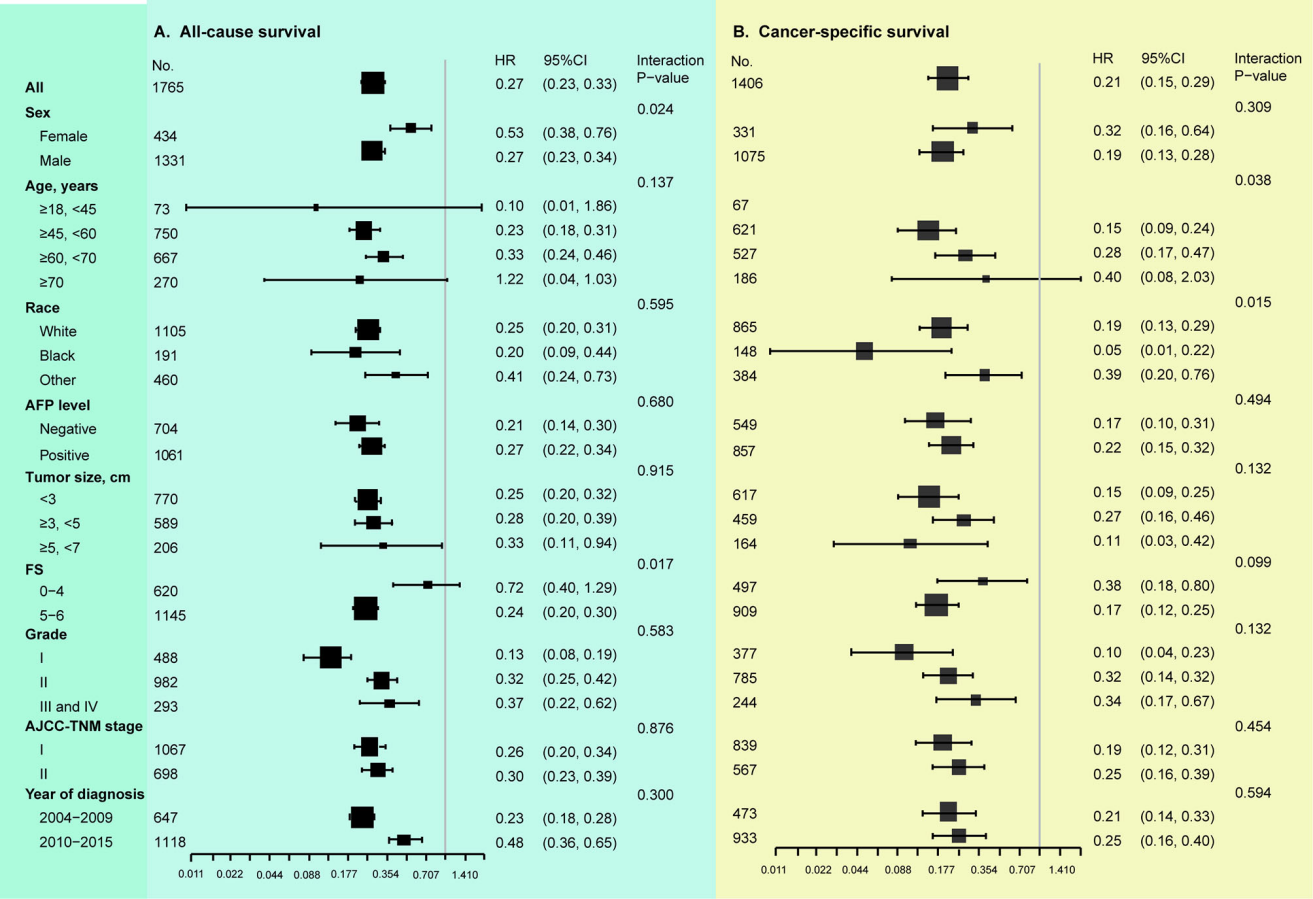
Stratified analysis based on clinicopathologic features (multivariable COX analyses). **(A)** Results of LT vs. LR on overall survival. **(B)** Results of LT vs. LR on disease-specific survival. AFP, alpha-fetoprotein; FS, fibrosis score; AJCC, American Joint Committee on Cancer. Tumor grade: I, well-differentiated; II, moderate-differentiated; III, poor-differentiated; IV, un-differentiated. In subgroup analysis, all identified confounders were adjusted except for the factor that the subgroup was based on.

Based on multivariable Cox analyses, the **Figure 4** showed the relation of surgical modality and patient prognosis stratified by clinical parameters. In subgroup analyses, the salutary effect of LT vs. LR on overall survival was consistent in all subgroups, except for a similar outcome in the non-cirrhotic subgroup (HR 0.72, 95%CI 0.40-1.29, interaction P value = 0.017) (**Figure 4A**). The superior survival benefits of LT vs. LR on DSS were consistent across all subgroups with the exception of a similar outcome in the subgroup of age > 70 years (HR 0.40, 95%CI 0.08-2.03, interaction P value = 0.038) (**Figure 4B**).

**Figure 5:**
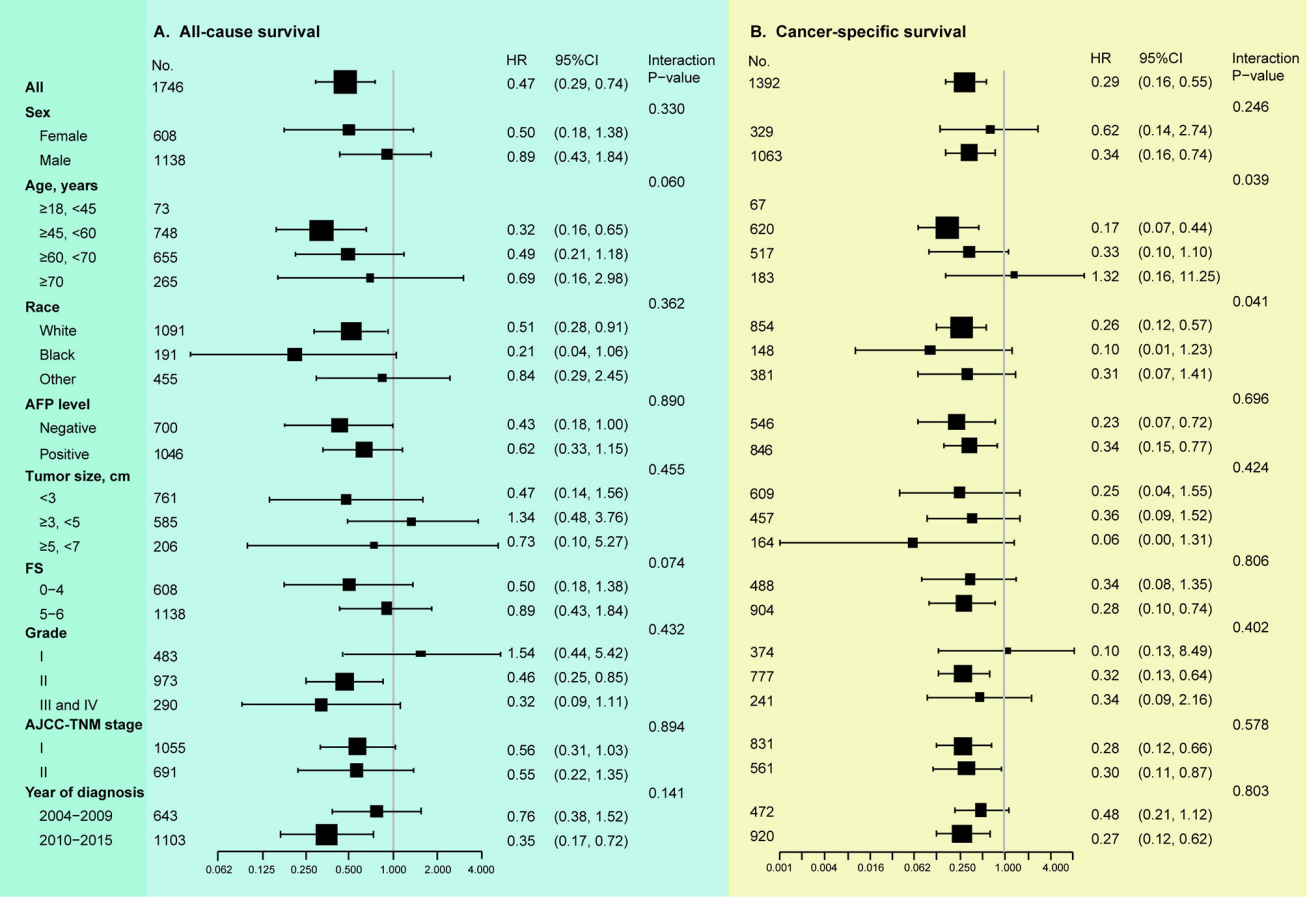
Stratified analyses according to clinicopathologic parameters (instrumental variable analyses). **(A)** Results of LT vs. LR on overall survival. **(B)** Results of LT vs. LR on disease-specific survival. AFP, alpha-fetoprotein; FS, fibrosis score; AJCC, American Joint Committee on Cancer. Tumor grade: I, well-differentiated; II, moderate-differentiated; III, poor-differentiated; IV, un-differentiated. In subgroup analysis, all identified confounders were adjusted except for the factor that the subgroup was based on.

In IV analyses stratified by clinical variables, we observed that the treatment effect of LT (OS) was consistent across all subgroups (all interaction P values > 0.05), as well as in those with a non-cirrhotic liver (**Figure 5A**). With regard to DSS, the exploratory subgroup analyses observed similar results in the older patients (> 60, <70 years: HR 0.33, 95%CI 0.10-1.10; ≥ 70 years: HR 1.32, 95%CI 0.16-11.25, interaction P value = 0.039) and the non-White population (Black: HR 0.10, 95%CI 0.01-1.23; Other: HR 0.31, 95%CI 0.07-1.41, interaction P value = 0.041), and LT had better DSS across the other subgroups (**Figure 5B**).

### Results in Propensity Score Matched Cohort

As presented in **Supplementary Table 2**, in the matched cohort, most of the prognostic variables were well-balanced. After PSM, cases receiving LT showed better DSS and OS (both P values < 0.001) compared to patients undergoing LR (**Figures 3B, D**). In the PSM cohort, the univariable analysis demonstrated that patients after LT still showed better DSS (HR 0.24, 95% CI 0.16-0.35, P < 0.001) and OS (HR 0.42, 95% CI 0.33-0.55, P < 0.001) in comparison to cases after LR (**Table 3**). In the Cox model only adjusting for propensity score, patients undergoing LT had both longer DSS (continuous: HR 0.23, 95% CI 0.17-0.32, P < 0.001; quintile: HR 0.26, 95% CI 0.18-0.36, P < 0.001) and OS (continuous: HR 0.41, 95% CI 0.32-0.52, P < 0.001; quintile: HR 0.43, 95% CI 0.34-0.54, P < 0.001) (**Table 3**).

**Table 3:** Association of surgical methods with patient overall survival.

	All-cause survival			Cancer-specific survival		
	Number	HR (95% CI)	P-value	Number	HR (95% CI)	P-value
Non-adjusted	1765	0.45 (0.38, 0.54)	<0.001	1,406	0.28 (0.21, 0.36)	<0.001
Multivariable adjusted model*	1765	0.27 (0.23, 0.33)	<0.001	1,406	0.21 (0.15, 0.29)	<0.001
Matched on propensity score**	778	0.42 (0.33, 0.55)	<0.001	616	0.24 (0.16, 0.35)	<0.001
Regression adjusted with propensity score						
Propensity score, continuous	1765	0.41 (0.32, 0.52)	<0.001	1,406	0.23 (0.17, 0.32)	<0.001
Propensity score, quintile	1765	0.43 (0.34, 0.54)	<0.001	1,406	0.26 (0.18, 0.36)	<0.001
Weighted models						
SMRW	1765	0.33 (0.28, 0.39)	<0.001	1,406	0.21 (0.16, 0.27)	<0.001
IPTW	1765	0.39 (0.33, 0.47)	<0.001	1,406	0.25 (0.20, 0.33)	<0.001

Data are shown as HR (95% CI) P value. *Adjusted model was adjusted for: race, age, sex, year of diagnosis, AFP level, tumor grade, fibrosis score, tumor size, and AJCC-TNM stage if available. **PSM model was based on the following variables: race, age, sex, year of diagnosis, AFP level, fibrosis score, tumor grade, tumor size, and AJCC-TNM stage. IPTW, inverse-probabilityof-treatment weighted; SMRW, standardized mortality ratio weighted.

### Outcomes in IPTW and SMRW Analyses

After propensity score reweighting using the IPTW method, tumor size remained imbalanced. All other parameters were well-balanced in SMRW (data not shown). As shown in **Table 3**, in the IPTW cohort, patients who underwent LT showed better OS (HR 0.39, 95% CI 0.33-0.47, P < 0.001) and DSS (HR 0.25, 95% CI 0.20-0.33, P < 0.001) in comparison to cases with LR (**Table 3**). In the SMRW cohort, patients with LT showed better DSS (HR 0.21, 95% CI 0.16-0.27, P < 0.001) and OS (HR 0.33, 95% CI 0.28-0.39, P < 0.001) in comparison to those after LR.

## Discussion

In the present study, we aimed to explore the independent role of surgical modality (LT *vs.* LR) in long-term survival for cases with curable stage I and II HCC. Both conventional multivariable regression analyses and the propensity score reweighting methods indicated that cases after LT had better DSS and OS in comparison to cases after LR. Additionally, when accounting for both the known and unknown confounders by IV analyses, LT still showed significant survival benefit compared to LR, whereas the adjusted coefficients were increased (the survival benefits were decreased). In stratified IV analyses, we found that non-White patients and patients with age ≥60 years undergoing LT had similar DSS compared to patients after LR.

Previous studies which compared the effectiveness of LT *vs.* LR have increased in the past decade (5, 6, 8, 11, 12, 20). However, the majority of studies comparing LT and LR for HCC were single-institutional, descriptive or retrospective comparisons. Conventional observational studies have utilized multivariable regression analysis and propensity score methods to evaluate associations between surgical modality and patient prognosis. However, these analyses could not adjust unmeasured confounders (15). In contrast, IV analysis allowed for an unbiased estimation of the treatment effect in cases whose treatment option varied with the instrument variable. The instrumental variable analysis was a type of quasi-experimental and econometric modality using naturally existing variation to produce pseudorandomization. Outcomes from IV analysis were found to be more similar to results from randomized controlled trials (RCTs) (15). IV analysis calculated the treatment effect on the marginal patients, while not the average treatment effect of LT (13, 18) thus, the IV analysis did not need to define the specific clinical characteristics of the populations. Instead, it was based on the precondition that cases resided randomly around hospitals and some cases were treated differently in distinct hospitals.

Milan criteria are the benchmark for selection of cases with HCC for LT and the reference for comparison with other criteria (1). For patients within stages I and II, some of them had HCC beyond the Milan criterion (*e.g.*, tumor diameter >5 cm). In subgroup analyses, we found that patients with tumor of 5–7 cm undergoing LT still had better OS compared to those after LR, which was consistent with some expanded criteria such as the Up-to-seven criteria (21) and Hangzhou criteria (22). Specially, in stratified analyses, patients with age >60 years after LT were found to have a similar long-term prognosis compared to those after LR. It was possibly because older patients have more medical comorbidities and poorer performance status. Chen et al. showed that the risk of death increased with an increase in the age at transplantation, especially in dialysis patients (23). Sharma et al. showed that cases aged 70 years and older had obviously higher mortality following LT (24). These observations along with our results should make surgeons aware of the necessity for better risk classification in elderly LT candidates. Especially, in IV analyses, we found that Non-white patients cannot acquire a better survival benefit after LT, which may be caused by the differences in environmental, cultural, social, and genetic factors between the White and non-White patients.

Admittedly, the current study had several limitations. First, some clinicopathologic data including preoperative liver function, comorbidities, performance status, postoperative morbidities, and postoperative treatments were not available in the SEER registry, thus we could not evaluate the impact of these factors on patient survival in multivariable analyses. Second, the observations of this study should be interpreted cautiously, given that a number of cases were excluded from our main analysis owing to the unavailable covariates in the SEER registry. Finally, even though IV analysis was a useful practical alternative to RCTs, its validity depended on the population studied. IV analyses only evaluated the effect on marginal patients, whereas patients who would always or never receive LT were excluded in the marginal cases, and it only focused on HCC cases with uncertain indications for LT.

Despite the increasing incidence of cases with HCC diagnosed at an earlier stage, LT rate decreased in the most recent era. By integrating multivariable analysis, PSM method and instrumental variable analysis, our results indicated that LT provided a survival benefit for marginal cases with stage I-II HCC. These results showed that if LT rates were to increase in the future, average survival time may also increase. However, for elderly patients, owing to the similar outcomes between LT and LR, the selection of LT should be cautious.

## Data Availability Statement

The raw data supporting the conclusions of this article will be made available by the authors, without undue reservation.

## Author Contributions

WL proposed the study. WL, HW, and YZ performed the research and wrote the first draft. WL collected and analyzed the data. HX revised this manuscript and validated the statistical methods of this study. YZ is the guarantor. All authors contributed to the design and interpretation of the study and to further drafts, and have read and approved the final version to be published.

## Conflict of Interest

The authors declare that the research was conducted in the absence of any commercial or financial relationships that could be construed as a potential conflict of interest.

## Supplementary Material

Supplementary Table 1 Characteristics of patients by quintile of Health Services Area LT rates.

Supplementary Table 2 Clinical features of the included patients with hepatocellular carcinoma after PSM.

## References

[1] EASL Clinical Practice Guidelines: Management of hepatocellular carcinoma. J Hepatol (2018) 69(1):182–236. 10.1016/j.jhep.2018.03.019 29628281

[2] Akinyemiju T Abera S Ahmed M Alam N Alemayohu MA Allen C. The Burden of Primary Liver Cancer and Underlying Etiologies From 1990 to 2015 At the Global, Regional, and National Level: Results From the Global Burden of Disease Study 2015. JAMA Oncol (2017) 3:1683–91. 10.1001/jamaoncol.2017.3055 PMC5824275 28983565

[3] Fonseca AL Cha CH. Hepatocellular Carcinoma: A Comprehensive Overview of Surgical Therapy. J Surg Oncol (2014) 110:712–9. 10.1002/jso.23673 24894746

[4] Forner A Llovet JM Bruix J. Hepatocellular Carcinoma. Lancet (2012) 379:1245–55. 10.1016/S0140-6736(11)61347-0 PMC13537732 22353262

[5] Baccarani U Isola M Adani GL Benzoni E Avellini C Lorenzin D. Superiority of Transplantation Versus Resection for the Treatment of Small Hepatocellular Carcinoma. Transplant Int Off J Eur Soc Organ Transplant (2008) 21:247–54. 10.1111/j.1432-2277.2007.00597.x 18028264

[6] Seshadri RM Besur S Niemeyer DJ Templin M McKillop IH Swan RZ. Survival Analysis of Patients With Stage I and II Hepatocellular Carcinoma After a Liver Transplantation or Liver Resection. HPB Off J Int Hepato Pancreato Biliary Assoc (2014) 16:1102–9. 10.1111/hpb.12300 PMC4253334 24964271

[7] Burman B Helton WS. Disparities in Care for Patients With Curable Hepatocellular Carcinoma. HPB Off J Int Hepato Pancreato Biliary Assoc (2015) 17:745–6. 10.1111/hpb.12477 PMC4557646 26278320

[8] Facciuto ME Rochon C Pandey M Rodriguez-Davalos M Samaniego S Wolf DC. Surgical Dilemma: Liver Resection or Liver Transplantation for Hepatocellular Carcinoma and Cirrhosis. Intention-to-treat Analysis in Patients Within and Outwith Milan Criteria. HPB Off J Int Hepato Pancreato Biliary Assoc (2009) 11:398–404. 10.1111/j.1477-2574.2009.00073.x PMC2742609 19768144

[9] Moon DB Lee SG Hwang S. Liver Transplantation for Hepatocellular Carcinoma: Single Nodule With Child-Pugh Class A Sized Less Than 3 Cm. Digest Dis (Basel Switzerland) (2007) 25:320–8. 10.1159/000106912 17960067

[10] Huang X Lu S. A Meta-analysis Comparing the Effect of Anatomical Resection vs. non-Anatomical Resection on the Long-Term Outcomes for Patients Undergoing Hepatic Resection for Hepatocellular Carcinoma. HPB Off J Int Hepato Pancreato Biliary Assoc (2017) 19:843–9. 10.1016/j.hpb.2017.06.003 28739076

[11] Menahem B Lubrano J Duvoux C Mulliri A Alves A Costentin C. Liver Transplantation Versus Liver Resection for Hepatocellular Carcinoma in Intention to Treat: An Attempt to Perform an Ideal Meta-Analysis. (2017) 23:836–44. 10.1002/lt.24758 28295992

[12] Benjamin AJ Baker TB Talamonti MS Bodzin AS Schneider AB Winschester DJ. Liver Transplant Offers a Survival Benefit Over Margin Negative Resection in Patients With Small Unifocal Hepatocellular Carcinoma and Preserved Liver Function. Surgery (2018) 163:582–6. 10.1016/j.surg.2017.12.005 29370929

[13] McDowell BD Chapman CG Smith BJ Button AM Chrischilles EA Mezhir JJ. Pancreatectomy Predicts Improved Survival for Pancreatic Adenocarcinoma: Results of an Instrumental Variable Analysis. Ann Surg (2015) 261:740–5. 10.1097/SLA.0000000000000796 PMC4277740 24979599

[14] Baiocchi M Cheng J Small DS. Instrumental Variable Methods for Causal Inference. Stat Med (2014) 33:2297–340. 10.1002/sim.6128 PMC4201653 24599889

[15] Terza JV Basu A Rathouz PJ. Two-Stage Residual Inclusion Estimation: Addressing Endogeneity in Health Econometric Modeling. J Health Economics (2008) 27:531–43. 10.1016/j.jhealeco.2007.09.009 PMC2494557 18192044

[16] Xu H Xia Z Jia X Chen K Li D Dai Y. Primary Tumor Resection Is Associated With Improved Survival in Stage Iv Colorectal Cancer: An Instrumental Variable Analysis. Sci Rep (2015) 5:16516. 10.1038/srep16516 26563729 PMC4643284

[17] Gore JL Litwin MS Lai J Yano EM Madison R Setodji C. Use of Radical Cystectomy for Patients With Invasive Bladder Cancer. J Natl Cancer Inst (2010) 102:802–11. 10.1093/jnci/djq121 PMC3245689 20400716

[18] Valley TS Sjoding MW Ryan AM Iwashyna TJ Cooke CR. Association of Intensive Care Unit Admission With Mortality Among Older Patients With Pneumonia. Jama (2015) 314:1272–9. 10.1001/jama.2015.11068 PMC4758179 26393850

[19] Tan HJ Norton EC Ye Z Hafez KS Gore JL Miller DC. Long-Term Survival Following Partial vs Radical Nephrectomy Among Older Patients With Early-Stage Kidney Cancer. Jama (2012) 307:1629–35. 10.1001/jama.2012.475 PMC3864575 22511691

[20] Kutlu OC Chan JA Aloia TA Chun YS Kaseb AO Passot G. Comparative Effectiveness of First-Line Radiofrequency Ablation Versus Surgical Resection and Transplantation for Patients With Early Hepatocellular Carcinoma. Cancer (2017) 123:1817–27. 10.1002/cncr.30531 28085184

[21] Mazzaferro V Llovet JM Miceli R Bhoori S Schiavo M Mariani L. Predicting Survival After Liver Transplantation in Patients With Hepatocellular Carcinoma Beyond the Milan Criteria: A Retrospective, Exploratory Analysis. Lancet Oncol (2009) 10:35–43. 10.1016/S0739-5930(09)79300-6 19058754

[22] Zheng SS Xu X Wu J Chen J Wang WL Zhang M. Liver Transplantation for Hepatocellular Carcinoma: Hangzhou Experiences. Transplantation (2008) 85:1726–32. 10.1097/TP.0b013e31816b67e4 18580463

[23] Chen HP Tsai YF Lin JR Liu FC Yu HP. Recipient Age and Mortality Risk After Liver Transplantation: A Population-Based Cohort Study. PloS One (2016) 11:e0152324. 10.1371/journal.pone.0152324 27019189 PMC4809564

[24] Sharma M Ahmed A Wong RJ. Significantly Higher Mortality Following Liver Transplantation Among Patients Aged 70 Years and Older. (2017) 27:225–31. 10.1177/1526924817715468 29187098

