# Supplementary material for: Liver Transplantation Versus Liver Resection for Stage I and II Hepatocellular Carcinoma: Results of an Instrumental Variable Analysis
Source: Front Oncol. 2021 May 26;11:592835. doi: 10.3389/fonc.2021.592835 (PMC8189150; doi:10.3389/fonc.2021.592835)
Supplement: Supplementary Table 1 — Characteristics of patients by quintile of Health Services Area LT rates. [file Table_1.docx]

| Supplementary Table 1. Characteristics of patients by quintile of Health Services Area LT rates. | | | | | |
| --- | --- | --- | --- | --- | --- |
| Variable | Area LT rate quintile | | | | |
|  | Quintile 1 (n=338) | Quintile 2 (n=336) | Quintile 3 (n=369) | Quintile 4 (n=284) | Quintile 5 (n=419) |
| LT rates in HSAs | 0.03 ± 0.00 | 0.04 ± 0.00 | 0.04 ± 0.00 | 0.05 ± 0.00 | 0.08 ± 0.02 |
| Age (years) | 60.4 ± 9.9 | 61.0 ± 10.3 | 60.0 ± 10.7 | 59.0 ± 9.7 | 60.3 ± 8.6 |
| Sex |  |  |  |  |  |
| Female | 77 (22.8%) | 104 (31.0%) | 82 (22.2%) | 63 (22.2%) | 104 (24.8%) |
| Male | 261 (77.2%) | 232 (69.0%) | 287 (77.8%) | 221 (77.8%) | 315 (75.2%) |
| Race |  |  |  |  |  |
| White | 175 (51.8%) | 212 (63.1%) | 197 (53.4%) | 192 (67.6%) | 315 (75.2%) |
| Black | 23 (6.8%) | 32 (9.5%) | 28 (7.6%) | 22 (7.7%) | 86 (20.5%) |
| Other | 137 (40.5%) | 90 (26.8%) | 144 (39.0%) | 67 (23.6%) | 17 (4.1%) |
| Unknown | 3 (0.9%) | 2 (0.6%) | 0 (0.0%) | 3 (1.1%) | 1 (0.2%) |
| AFP (ng/ml) |  |  |  |  |  |
| Negative | 134 (39.6%) | 130 (38.7%) | 164 (44.4%) | 110 (38.7%) | 162 (38.7%) |
| Positive | 204 (60.4%) | 206 (61.3%) | 205 (55.6%) | 174 (61.3%) | 257 (61.3%) |
| Fibrosis score |  |  |  |  |  |
| 0-4 | 144 (42.6%) | 145 (43.2%) | 134 (36.3%) | 78 (27.5%) | 107 (25.5%) |
| 5-6 | 194 (57.4%) | 191 (56.8%) | 235 (63.7%) | 206 (72.5%) | 312 (74.5%) |
| Tumor size (mm) | 41.4 ± 34.0 | 44.7 ± 34.7 | 42.4 ± 54.3 | 40.0 ± 36.3 | 36.8 ± 29.8 |
| AJCC-TNM stage |  |  |  |  |  |
| I | 212 (62.7%) | 211 (62.8%) | 222 (60.2%) | 159 (56.0%) | 251 (59.9%) |
| II | 126 (37.3%) | 125 (37.2%) | 147 (39.8%) | 125 (44.0%) | 168 (40.1%) |
| Tumor differentiation |  |  |  |  |  |
| I | 114 (33.7%) | 82 (24.4%) | 120 (32.5%) | 50 (17.6%) | 117 (27.9%) |
| II | 166 (49.1%) | 183 (54.5%) | 192 (52.0%) | 193 (68.0%) | 239 (57.0%) |
| III | 53 (15.7%) | 67 (19.9%) | 52 (14.1%) | 38 (13.4%) | 62 (14.8%) |
| IV | 5 (1.5%) | 4 (1.2%) | 5 (1.4%) | 3 (1.1%) | 1 (0.2%) |
| Data are shown as mean ± SD or n (%). LT, liver transplantation; HSA, Health Service Area; AFP, alpha-fetoprotein; AJCC, American Joint Committee on Cancer. Tumor differentiation: I, well-differentiated; II, moderate- differentiated; III, poor-differentiated; IV, un-differentiated. | | | | | |
|  |  |  |  |  |  |
