# Supplementary material for: Liver Transplantation Versus Liver Resection for Stage I and II Hepatocellular Carcinoma: Results of an Instrumental Variable Analysis
Source: Front Oncol. 2021 May 26;11:592835. doi: 10.3389/fonc.2021.592835 (PMC8189150; doi:10.3389/fonc.2021.592835)
Supplement: Supplementary Table 2 — Clinical features of the included patients with hepatocellular carcinoma after PSM. [file Table_2.docx]

| Supplementary Table 2. Clinical features of the included patients with hepatocellular carcinoma after PSM. | | | | |
| --- | --- | --- | --- | --- |
|  | LR (n=389) | LT (n=389) | SD | P value |
| Age (years) | 59.57 ± 9.01 | 58.77 ± 7.55 | 0.095 | 0.184 |
| Sex |  |  | 0.000 | 1.000 |
| Female | 97 (24.9) | 97 (24.9) |  |  |
| Male | 292 (75.1) | 292 (75.1) |  |  |
| Race |  |  |  | 0.630 |
| White | 247 (63.5) | 259 (66.6) | 0.065 |  |
| Black | 52 (13.4) | 42 (10.8) | 0.079 |  |
| Other | 89 (22.9) | 86 (22.1) | 0.019 |  |
| Unknown | 1 (0.3) | 2 (0.5) | 0.042 |  |
| Year of diagnosis |  |  | 0.012 | 0.373 |
| 2004-2009 | 150 (38.6%) | 138 (35.5%) |  |  |
| 2010-2015 | 239 (61.4%) | 251 (64.5%) |  |  |
| AFP (ng/ml) |  |  | 0.043 | 0.600 |
| Negative | 134 (34.4) | 142 (36.5) |  |  |
| Positive | 255 (65.6) | 247 (63.5) |  |  |
| Tumor size (cm) | 30.85 ± 12.98 | 29.74 ± 13.41 | 0.084 | 0.240 |
| AJCC-TNM stage |  |  | 0.026 | 0.770 |
| I | 237 (60.9) | 232 (59.6) |  |  |
| II | 152 (39.1) | 157 (40.4) |  |  |
| Fibrosis score |  |  | 0.013 | 0.929 |
| 0-4 | 80 (20.6) | 78 (20.1) |  |  |
| 5-6 | 309 (79.4) | 311 (79.9) |  |  |
| Tumor differentiation |  |  |  | 0.600 |
| I | 105 (27) | 119 (30.6) | 0.078 |  |
| II | 219 (56.3) | 212 (54.5) | 0.036 |  |
| III | 61 (15.7) | 56 (14.4) | 0.036 |  |
| IV | 4 (1) | 2 (0.5) | 0.059 |  |
| Data are shown as mean ± SD or n (%). LR, liver resection; LT, liver transplantation; SD, standard deviation; AFP, alpha-fetoprotein; AJCC, American Joint Committee on Cancer. Tumor differentiation: I, well-differentiated; II, moderate- differentiated; III, poor-differentiated; IV, un-differentiated. | | | | |
|  |  |  |  |  |
|  |  |  |  |  |
